# Global Trends in Research of Pain–Gut-Microbiota Relationship and How Nutrition Can Modulate This Link

**DOI:** 10.3390/nu15173704

**Published:** 2023-08-24

**Authors:** Gaochen Lu, Sheng Zhang, Rui Wang, Zulun Zhang, Weihong Wang, Quan Wen, Faming Zhang, Pan Li

**Affiliations:** 1 Department of Microbiota Medicine, Medical Center for Digestive Diseases, The Second Affiliated Hospital of Nanjing Medical University, Nanjing 210011, China; lugaochen@njmu.edu.cn (G.L.); zslyre@163.com (S.Z.); brucewong_wr@126.com (R.W.); zulunzhang@stu.njmu.edu.cn (Z.Z.); wwhome0703@163.com (W.W.); wenquan@njmu.edu.cn (Q.W.); 2 Key Lab of Holistic Integrative Enterology, Nanjing Medical University, Nanjing 210011, China; 3 Department of Microbiotherapy, Sir Run Run Hospital, Nanjing Medical University, Nanjing 211166, China; 4 National Clinical Research Center for Digestive Diseases, Xi’an 710032, China

**Keywords:** bibliometric, gut, microbiota, nutrition, pain, microbiota medicine

## Abstract

Introduction: The link between gut microbiota and chronic painful conditions has recently gained attention. Nutrition, as a common intervention in daily life and medical practice, is closely related to microbiota and pain. However, no published bibliometric reports have analyzed the scientific literature concerning the link. Methods and results: We used bibliometrics to identify the characteristics of the global scientific output over the past 20 years. We also aimed to capture and describe how nutrition can modulate the abovementioned link. Relevant papers were searched in the Web of Science database. All necessary publication and citation data were acquired and exported to Bibliometrix for further analyses. The keywords mentioned were illustrated using visualization maps. In total, 1551 papers shed light on the relationship from 2003 to 2022. However, only 122 papers discussed how nutritional interventions can modulate this link. The citations and attention were concentrated on the gut microbiota, pain, and probiotics in terms of the pain–gut relationship. Nutritional status has gained attention in motor themes of a thematic map. Conclusions: This bibliometric analysis was applied to identify the scientific literature linking gut microbiota, chronic painful conditions, and nutrition, revealing the popular research topics and authors, scientific institutions, countries, and journals in this field. This study enriches the evidence moving boundaries of microbiota medicine as a clinical medicine.

## 1. Introduction

Pain, an unpleasant sensory and emotional experience, is subjective and involves not only nociception but also emotional, cognitive, and social components [1]. Compared with acute pain, which serves as an alarm system to protect us from tissue damage, chronic pain is very disturbing and can reduce quality of life [2,3]. While we know that peripheral organs such as the skin, muscle, bones, joints, and even deep visceral tissues are populated locally with nociceptors, the molecular and cellular mechanisms of chronic pain are not fully understood. Chronic pain includes pathological pain, psychogenic pain, functional pain, etc. The pain mentioned in this article does not equal discomfort. Discomfort is a milder, non-specific feeling of unease, whereas pain is a more intense and specific sensation that typically indicates potential harm or injury to the body. Clinically, there is still a shortage of safe and effective therapeutic approaches to the management of chronic pain.

Types of chronic pain include visceral, inflammatory, headache, and neuropathic pain, and treatments vary according to type [4,5]. Visceral pain related to the abdominal and back regions, such as the pain of Crohn’s disease (CD), is always a clinical challenge for a physician. A systematic review found that no conclusions could be drawn about the efficacy of the majority of interventions for CD (e.g., low FODMAP diet, kefir diet, acupuncture, stress relief, enteric-soluble nitroglycerin, olorinab, relaxation training, and yoga) in treating pain intensity and pain frequency, with the exception of transcranial direct current stimulation [6]. Similarly, treatment for neuropathic pain, such as that caused by distal symmetric polyneuropathy (DSPN), the most common neuropathy in patients with diabetes mellitus (DM), is a pernicious unmet medical need [7,8]. Although neuropathic pain and lower-limb amputations due to DSPN negatively affect patients’ functionality, health-related quality of life, and mortality, glucose control and a few medications may only have limited effect on these symptoms [9].

The gut microbiota is the most complex and populous ecosystem in our body [10]. The homeostasis between gut microbiota and the host is important for the maintenance of health, specifically including gut-barrier integrity, protection from pathogens, energy regulation, nutrition storage, brain development, and immune homeostasis. Given the increasingly recognized potential of gut microbiota, their role in the regulation of pain has been attracting more attention recently; probiotics, prebiotics, synbiotics, postbiotics, and fecal microbiota transplantation (FMT) are potential technologies to regulate gut microbiota [11,12,13,14,15,16]. As such, microbiota, which colonize in the gastrointestinal tract, can mediate the bidirectional communication between the gut and pain through interaction between bacteria and their composition or metabolites, such as short-chain fatty acids (SCFAs), bile acids (BAs), and tryptophan metabolites. Endocrine (adrenaline, cortisol, Ach, etc.), immune (immune cell, cytokine, etc.), and neural (vagus nerve, enteric nervous system, etc.) are the major pathways here [17].

Nutrition is the biological process of taking in and utilizing food nutrients to maintain normal physiological, biochemical, and immune functions, as well as life activities such as growth, metabolism, and repair. Nutritional disorders are the result of an inadequate balance between energy intake and energy expenditure; this balance plays an important role in the occurrence, development, treatment, and prognosis of many diseases [18]. Refractory malnutrition has always been a serious challenge for hospitals, patients, and society, and can even lead to disability and death. Indigestible macronutrients from the diet can be metabolized by healthy gut microbiota, resulting in SCFAs, BAs, and other bioactive compounds through digestion. The differences of gut microbiota and changeful SCFAs, BAs, and tryptophan metabolite levels, together with functional receptors or a signaling pathway, are closely related to chronic painful conditions, intestinal function, or nutritional status. Since diet has an influence on the composition of gut microbiota, nutritional disorders can be linked to an alteration thereof, mirroring the physiopathology.

We therefore turned our attention to the interaction between gut microbiota and pain and how nutrition can modulate this link. The most rapid and general approach to gaining an understanding of a field of research literature is performing a bibliometric analysis. Bibliometrics is an analytical approach that generates an integrative view and quantitative parameter profiling of specific scientific application areas or entire research fields [19,20]. Here, a bibliometric analysis was applied to identify the scientific literature on the links between gut microbiota, chronic painful conditions, and nutrition, revealing the popular research topics and the key authors, scientific institutions, countries, and journals. We retrieved publications published from 2003 to 2022 and their recorded information from the Science Citation Index Expanded (SCI-expanded) of the Web of Science Core Collection (WoSCC). To the best of our knowledge, this is the first total-scale bibliometric analysis of the scientific literature examining pain, gut microbiota, and nutrition in medicine. This study encourages physicians and researchers to comprehensively pay attention to the gut-microbiota–pain–nutrition axis and meets the scientific explanations of microbiota medicine as a branch discipline in clinical medicine [21].

## 2. Materials and Methods

### 2.1. Data Sources and Search Strategies

The Web of Science Core Collection (WoSCC) database is the largest academic information resource in the world, including more than 8700 core academic journals in various research fields, and was the most widely used tool for the bibliometric analysis. The references on pain–gut-microbiota–nutrition from January 2003 to December 2022 were identified. The WoSCC database was searched on a single day (1 March 2023) to avoid deviations. The two parts of search terms were presented as follows: Section 1: TS = (microbiota OR microbiome) AND TS = (pain OR ache). Section 2: TS = (microbiota OR microbiome) AND TS = (pain OR ache) AND TS = (nutrition* OR nutrient). The search terms identified publications that mention these words and their derivatives in the title, abstract, and recurring keywords. Only original articles and reviews written in English were included among various publication types. The research objects occurring in the literature extracted using the bibliometric analysis include humans, macaques, different types of mice and rats, pigs, and cows.

### 2.2. Data Evaluation

In this study, we used several indicators to evaluate these data from the WoSCC database as follows. The total number of publications (NP) and citations (NC) without self-citations, as two of the most basic indicators, were used to measure productivity and the overall impact. Citations per publication (CPP) were identified as the ratio of the above two (NC/NP) to represent the relative impact in the current area. To analyze CPP differences between original articles and reviews, a non-parametric test was conducted. Statistical tests were performed with SPSS (version 26.0; IBM Corp, Armonk, NY, USA). Results were deemed significant if *p* < 0.05.

In addition, the H-index, defined as the maximum value of h such that the given author or journal has published at least h papers that have each been cited at least h times, is used to evaluate the academic contribution, and predict future scientific achievements (https://en.wikipedia.org/wiki/H-index, accessed on 1 March 2023). Usually, the H-index is calculated from the dataset within the last 10 years. Indeed, the M-index, based on the H-index, is defined as the ratio of the H-index to the academic age since an author’s first publication, to avoid the influence of time.

Moreover, the local citations score (LC) and global citations score (GC) were used to describe the number of the given publications cited in this perspective and the whole WoSCC database. The ratio of both (LC/GC) was used to evaluate the importance of the given publication from this perspective. Similarly, the single-country publication (SCP) and multiple-country publication (MCP) were used to record the number of whether the corresponding author(s) are from a single country or multiple countries in the given country’s publications. The ratio (MCP/(MCP + SCP)) was used to reflect the cooperation of the given country.

### 2.3. Data Analysis and Visualization

The full records of the resultant publications were exported to VoSviewer (version 1.6.18, Leiden, The Netherlands) [22] and the package ‘Bibliometrix’ (version 4.0, operated under the web interface called Biblioshiny) [23] based on R software (version 4.2.2) for further bibliometric analyses. In total, 1551 papers in part 1 and 122 papers in part 2 were ultimately analyzed in our study.

Using the R package ‘Bibliometrix’, we performed a preliminary analysis of the overall information, including authors, journals, institutions, and the literature. Then, we calculated the corresponding indicators mentioned above to evaluate the productivity, academic contribution, and impact of authors, journals, and organizations, to measure the cooperation situation among countries, and to obtain the milestone literature concerning the publications in related research fields.

For a visual analysis, we calculated the correlation between the keywords and keywords plus, and then a thematic map concerning the keywords was drawn to discover the evolution of themes. On the thematic map, each bubble represents the top three clusters of keywords, while the size of the bubble indicates the number of keywords in the cluster. And the location of each bubble also has a different meaning; the closer to the right the bubble is, the more relevant the theme is, and the higher the bubble is, the more booming the theme is. As a result, a thematic map is divided into areas, with upper-left for niche themes, upper-right for motor themes, lower-left for emerging or declining themes, and lower-right for basic themes.

Collaborative networks between organizations and authors, and the co-occurrence of keyword clusters, involved the use of VoSviewer. On the network plotted with VoSviewer, different bubbles represent different elements, while the size of each bubble indicates the NP or co-occurrence frequency, where a larger NP/frequency means a larger size of its bubbles. In addition, the color of each bubble indicates the different clusters or times. A line between two bubbles reflects the relationship, where the larger the size of the cooperation, the thicker the connecting line.

## 3. Results

### 3.1. An Overview of Publications

**Figure 1:**
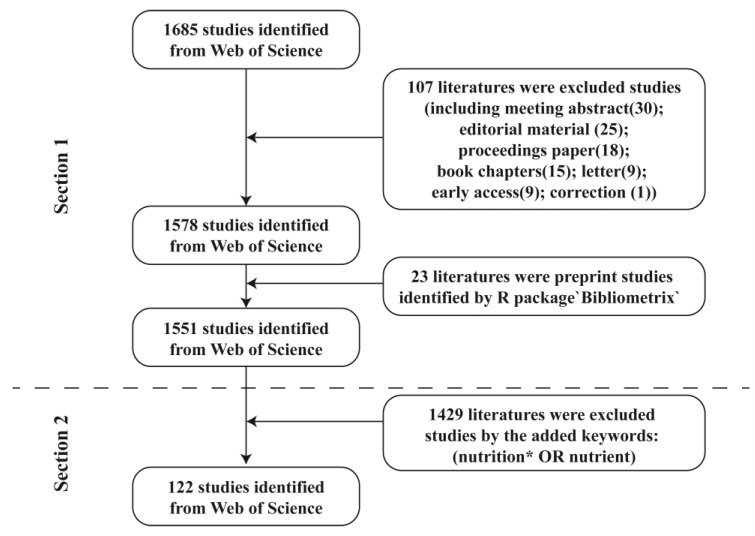
Flowchart of the screening process.

**Figure 2:**
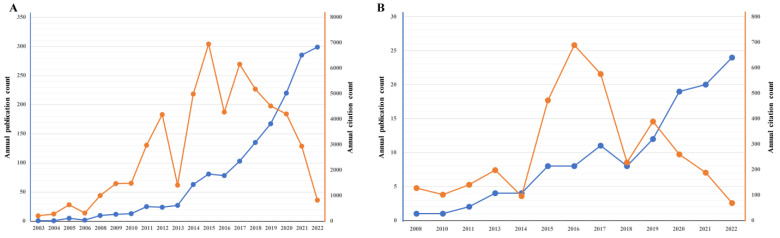
(**A**) Annual publication and citation count of research on the pain–gut-microbiota relationship. (**B**) Annual publication and citation count of research on the pain–gut-microbiota–nutrition relationship. The line in blue represents the annual publication count while the line in orange represents the annual citation count.

As shown in the flowchart (Figure 1), based on the Section 1 search strategy, 1551 publications from 2003 to 2022 focusing on the pain–gut-microbiota relationship were identified. From the earliest publication in 2003, the trend increased every year, reaching 100 publications in 2017 (Figure 2A). There were 1020 original articles (1020/1551; 65.76%; CPP = 29.59) and 531 reviews (531/1551; 34.24%; CPP = 45.00). Thus, the article-to-review ratio was 1.92:1. The reviews had a significantly higher CPP than the original articles (*p* = 0.003). The first publication about how nutrition can modulate the pain–gut-microbiota relationship came in 2008 (Figure 2B). In Section 2, the article-to-review ratio was 1.26:1. There were 68 original articles (68/122; 55.74%; CPP = 33.28) and 54 reviews (54/122; 44.26%; CPP = 23.37), and the articles had a higher CPP than reviews but no statistical difference (*p* = 0.76). However, the annual citation counts were fluctuant and not consistent with the publication counts.

### 3.2. Most Productive Entities in Recent Ten Years

The top ten most productive authors calculated with the number of publications are listed in Table 1. A total of 8292 authors participated in pain–gut-microbiota-related studies. The most productive author was Professor Cryan JF from APC Microbiome Ireland (one of the most famous institutions in the field of microbiome research); he also had the highest H-index and M-index among the top ten most productive authors, demonstrating his leading position in this field. Meanwhile, the author with the highest CPP was Professor Dinan TG, also from APC Microbiome Ireland. According to the M-index, the most impactful researchers in this field are still Cryan JF and Dinan TG. A total of 778 authors referred to how nutrition can modulate the link between pain and gut microbiota in their contributions (Table 1). Professor Simren M was the most productive author in this field. Although Mayer EA only published three articles related to nutrition, his high CPP indicated his relative and unmatched impact in this area recently. Meanwhile, Van Oudenhove L and Bercik P entered this area not until 2021, but they may be future stars for their high M-index.

The top ten most productive organizations are listed in Table 2, six of which are from the United States. The Mayo Clinic was the most productive organization and had the highest H-index, but the University of California, Los Angeles had a higher CPP than the Mayo Clinic. From the view of the M-index, Harvard Medical School was the top. The remaining four organizations were from Ireland, Canada, Sweden, and China, respectively. Nanjing Medical University, as the only one of the top ten institutions in Asia in this research area, also showed an excellent M-index. Taking nutrition intervention into consideration, the distribution of the top ten organizations was different. The United States was still the most productive organization. The Lerner Research Institute had the highest CPP among these organizations, despite having just three publications. Indeed, the University College Cork ranked first not only in the H-index but also in the M-index.

In general, the top ten most productive countries are shown in Table 3. The United States contributed nearly 22.95% of the publications on the pain–gut-microbiota relationship, followed by China with 18.25%. The international collaboration rates of the countries listed were mostly around 20–40%, with China and South Korea having lower rates of 11.31% and 9.09%, respectively. When nutrition intervention was considered, the United States still came out on top (25.41%), followed by Italy (13.93%) and China (7.38%). Although the United States had the most publications, the highest CPP belonged to Germany (Section 1 and Section 2, respectively).

The top ten most productive journals are listed in Table 4. Medicine was the leading area of research in these productive journals and seemed to have a much higher CPP compared to the other areas. Multidisciplinary sciences followed as the second most important, with a reasonably high CPP.

### 3.3. Popular Research Themes

**Figure 3:**
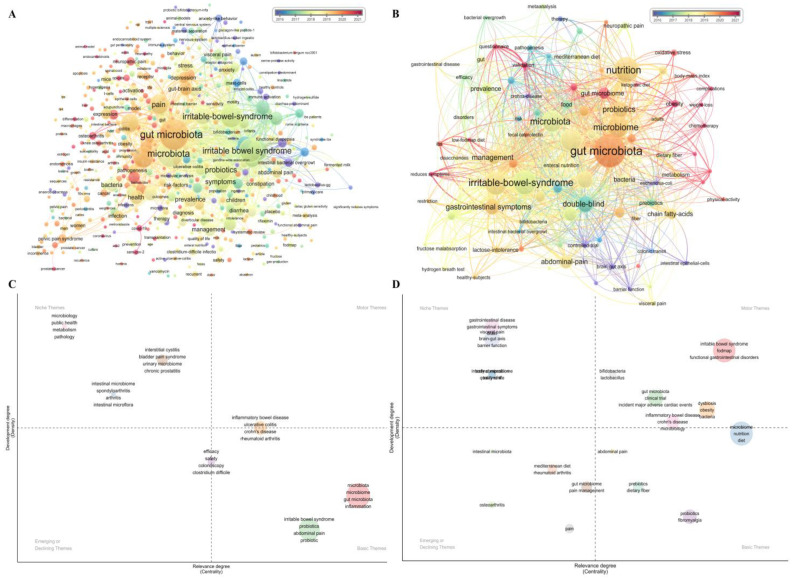
(**A**) Co-occurrence network of keywords plus with emerging time in the pain–gut-microbiota relationship. (**B**) Co-occurrence network of keywords plus with emerging time in the pain–gut-microbiota–nutrition relationship. The size of the bubble indicates the co-occurrence frequency, and the color of the bubble indicates different times. A line between bubbles reflects the strength of the links. (**C**) The thematic maps of evolution in keywords in the pain–gut-microbiota relationship. (**D**) The thematic maps of evolution in keywords in the pain–gut-microbiota–nutrition relationship. Each bubble represents the top three of the keywords’ clusters and its size indicates the number of keywords in the cluster. The closer to the right the bubble is, the more relevant the theme is; the higher the bubble is, the more booming the theme is.

VoSviewer was used to generate a term map targeting phrases mentioned in the keywords, titles, and abstracts of the publications with their emerging time (Figure 3A,B). Phases of keywords located in the center had more connections and occurrences. The average published year (APY) of these keywords was 2018, and the occurrences of keywords concerning microbiota ranked at the top regardless of context (the pain–gut-microbiota or pain–gut-microbiota–nutrition relationship).

In the context of the pain–gut-microbiota relationship (Figure 3A), 14.78% of keywords (620/6636) had over five times of co-occurrences. The top three keywords were “gut microbiota” (405/1551; APY = 2019.41; CPP = 24.57), “microbiota” (351/1551; APY = 2018.36; CPP = 29.38), and “irritable bowel syndrome” (260/1551; APY = 2017.87; CPP = 31.88). As one of the search terms, the general concept of the keyword “pain” ranked ninth (182/1551; APY = 201,916; CPP = 32.33) in the co-occurrences. Among the subclasses of “pain”, there were mainly 201 papers covering abdominal pain, 61 papers for pelvic and bladder pain, 59 papers for general visceral pain, as well as 43 papers for neuropathic pain and headache. In addition, COVID-19 was mentioned 21 times in the research (21/1551; APY = 2021.00; CPP = 16.52), thereby occurring in the top 1.75% of keywords.

In the context of nutrition intervention and the pain–gut relationship, 9.62% of the 1060 keywords had over three times of co-occurrences (Figure 3B). Meanwhile, although the keyword “gut microbiota” had the most occurrences (34/122), its APY was 0.41 years later and its CPP declined to only 22.62. The other top three keywords were “nutrition” (25/122; APY = 2019.12; CPP = 14.68) and “irritable bowel syndrome” (24/122; APY = 2018.04; CPP = 29.5). The rank of the general search term “pain” also declined to 29th (7/122; APY = 2019.86; CPP = 24.00). Among the subclass of “pain”, there were 12 papers covering abdominal pain, 6 papers for neuropathic pain, and 4 papers for general visceral pain.

Bibliometrix was used to explore the evolution of themes via the thematic map. In terms of the pain–gut-microbiota relationship (Figure 3C), the cluster of “inflammatory bowel disease”, “ulcerative colitis”, “Crohn’s disease”, and “rheumatoid arthritis” was the middle of motor themes (both important and booming) and basic themes. The cluster of “irritable bowel syndrome”, “probiotic(s)”, and “abdominal pain” together with the cluster of “gut microbiota”, “inflammation”, “microbiome”, and “microbiota” were two clusters of the basic themes, while the cluster of “safety”, “efficacy”, “*Clostridioides difficile*”, and “colonoscopy” was half in the emerging or declining area. Three clusters were in the field of the niche themes (not important but booming), such as the cluster of “public health”, “urinary microbiome”, and “metabolism”.

After we attached nutrition and the pain–gut-microbiota relationship, different results are shown in Figure 3D. Firstly, newer clusters are presented in the thematic map, such as the cluster of “*bifidobacteria*”, “*lactobacillus*”, and “fodmap” in motor themes, while a cluster of “brain–gut axis”, “visceral pain,” and “quality life” was in niche themes. The cluster of “pain management” was in emerging or declining themes, and a cluster of “nutrition” and “diet” was observed in the basic themes. Secondly, among others, the cluster containing “irritable bowel syndrome” went from basic to motor themes.

### 3.4. Collaborative Network Analysis of Entities

**Figure 4:**
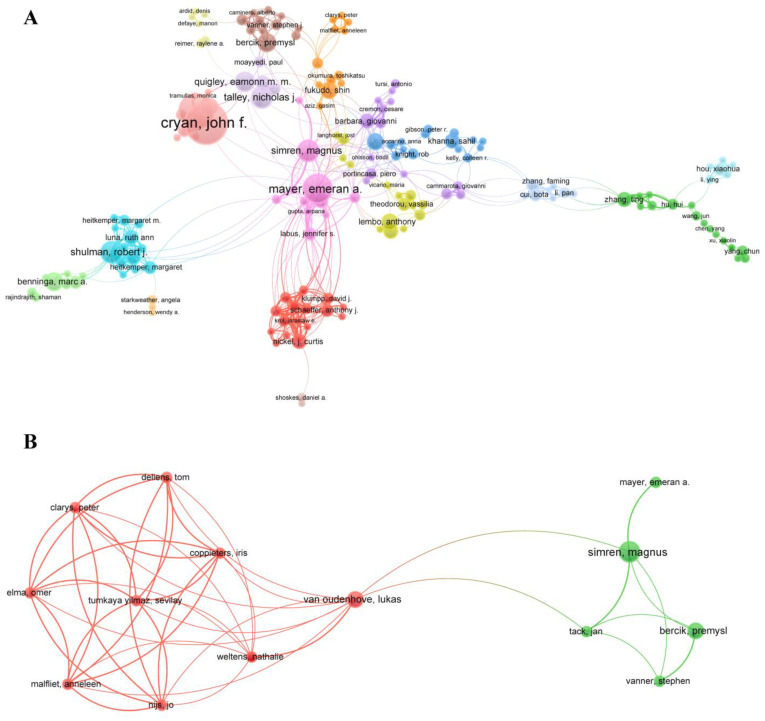
(**A**) Co-authorship network of authors in the pain–gut-microbiota relationship. (**B**) Co-authorship network of authors in the pain–gut-microbiota–nutrition relationship. The size of the bubble indicates the number of publications, and the color of the bubble indicates different clusters. A line between bubbles reflects the strength of the links.

The collaborative networks between authors are shown in Figure 4. In terms of the pain–gut-microbiota relationship, 1.34% authors (276/8937) collaborated with others more than three times. About 61.59% of 276 authors (170/276) formed a big central network of links and were divided into 17 clusters (Figure 4A). The cluster centered on Emeran A. Mayer had the most links with others, forming the main body of the network. However, Cryan JF, the second greatest link strength, had the most publications and citations worldwide in this field. In addition, in only 3 of the 17 clusters were the majority of members from China, such as the cluster centered on Faming Zhang, who had the most publications among the Chinese clusters. Indeed, in the context of the pain–gut-microbiota–nutrition relationship, 5.33% of the authors (42/788) worked with others more than two times. The center network here consisted of 14 authors and two clusters (Figure 4B). Authors in the same cluster were in close contact with each other within the cluster. The bridge between two clusters was Magnus Simren, Lukas V. Oudenhove, and Jan Tack in this field.

**Figure 5:**
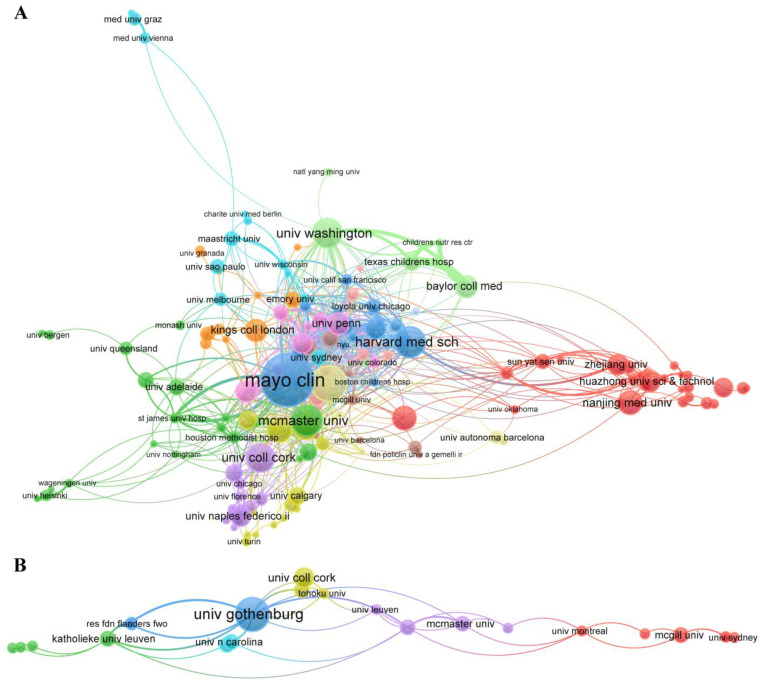
(**A**) Co-authorship network of organizations in the pain–gut-microbiota relationship. (**B**) Co-authorship network of organizations in the pain–gut-microbiota–nutrition relationship. The size of the bubble indicates the number of publications, and the color of the bubble indicates different clusters. A line between bubbles reflects the strength of the links.

The collaborative networks of the organizations are plotted in Figure 5. A total of 2408 organizations were involved in the pain–gut-microbiota relationship, but only 7.1% of these (171/2408) worked with each other over five times. The huge center network was made up of 164 organizations and 13 clusters (Figure 5A). The total link strength exceeded 60 for several organizations, including the Mayo Clinic, Harvard Medical School, and University of California, Los Angeles. Moreover, the organizations with the most publications in this field in America and China were the Mayo Clinic and Nanjing Medical University, respectively. Taking nutrition into account, 13.78% organizations (50/363) collaborated with others more than twice, while only 24 organizations and six clusters formed the center network (Figure 5B) of this pain–gut-microbiota–nutrition relationship. The cluster centered on the University of Gothenburg had the largest number of links and the greatest total link strength in this field.

### 3.5. Cited Literature Analysis

The top ten locally cited pieces of literature are listed in Table 5. Cryan et al. published in Nature Reviews Neuroscience in 2012, earning the most local and global citations and showing its great contributions to all WoSCC datasets [24]. In addition, Crouzet et al. had the highest ratio of LC/GC, due to this paper’s relative significance to this field in 2013 (Table 5 and Section 1) [25]. Unfortunately, the LC of the pieces of top 10 research, which involved the keyword “nutrition” in the pain–gut-microbiota relationship, was usually one. Enck et al. published in Nature Reviews Disease Primers in 2016, ranking first with the local citations and global citations [26]. However, the highest ratio of LC/GC involved the article published by Croisier et al. in the Journal of the Academy of Nutrition and Dietetics in 2021 (Table 5 and Section 2) [27].

## 4. Discussion

This study was the first bibliometric and visual analysis of research on the pain–gut-microbiota relationship and how nutrition can modulate this link, from January 2003 to December 2022 via the R package “Bibliometrix” and “VoSviewer”. In this manuscript, we pay attention to “pain”, rather than “discomfort”. Discomfort and pain are related sensations but have distinct differences. Our team is currently working on an integrated treatment plan for malnutrition (especially refractory malnutrition throughout patients’ life cycle) from a microecological perspective, which changes the diagnosis and treatment mode from Multi-Disciplinary Treatment (MDT) to Holistic Integrative Medicine (HIM). However, we find the overlooked role of nutrition when applying microbial therapy in a clinic. The analysis based on bibliometric tools casts new light on evolving research foci and trends, and this type of data analysis was relatively more comprehensive and objective compared to a traditional literature review.

### 4.1. Principal Findings

This study included 1551 articles on the pain–gut-microbiota relationship from the WoSCC database, of which 122 also involved the keyword “nutrition”. These articles started to come out in the 2000s, grew in number around 2008, and boomed from 2016 regarding the pain–gut-microbiota relationship. As shown in Figure 2B, publications on the pain–gut-microbiota–nutrition relationship started to be published in 2008, grew in 2013, and have been well developed since 2019. In a word, more attention has increasingly been paid to both these fields, which is reflected in the increasing rates of publication. In addition, there were two huge inflection points observed in the citations in Figure 2A,B. The first point was located in 2013–2014 for the consistence with the commercialization time of the third-generation sequencing technology. Most researchers sought a new research angle via novel technology and thus a boom in citations resulted in the later years. Due to the COVID-19 pandemic, another point was shown in 2018.

In terms of countries, the United States, one of the main driving forces of research in the world, contributed the most publications in both fields, followed by China and Italy. However, despite their high productivity of publications, the rate of international collaboration in both fields was very low between these countries; even a little cooperation in research between countries counts for much impact. Indeed, we should acknowledge that some countries, such as the United States, have sufficient research foundations and an internal academic atmosphere. Not only are countries in need of more research contact with others, as shown in Figure 4 and Figure 5, but organizations and authors also have this need too. In the field of pain and gut microbiota, only 1.34% of authors collaborated with each other more than three times. In total, 7.1% of organizations worked with others more than five times. These shed some light on the current situation: many researchers and organizations prefer to pursue their aims alone or collaborate with small internal groups. The same trend was found in pain–gut-microbiota–nutrition.

Additionally, the H-index and M-index are used to evaluate the academic contribution and predict future scientific achievements. Cryan JF and Dinan TG, who had the highest H-index, should be considered the most influential authors in the last decade in the field of the pain–gut-microbiota relationship. Even excluding the factor of the year of publication, both Cryan JF and Dinan TG had the highest M-index, indicating a high and stable contribution to this field. With nutrition added into the mix, Polivka J may be a potential future high-impact author regarding the pain–gut-microbiota–nutrition relationship due to his high M-index. When it comes to organizations, the National University of Ireland had the highest impact in the field of the pain–gut-microbiota relationship over the last ten years, while Nanjing Medical University’s impact was becoming more stable over these years due to the highest M-index in this field. Finally, most organizations in the field of pain–gut-microbiota–nutrition had a low M-index and publications. The shorter emergence time may partly explain this phenomenon, but more efforts need to be put into developing the field.

All the keywords in the thematic map were put into different areas. The motor themes, located in the upper-right area, represent importance and boom while the lower left-area represents emerging or declining themes. As traditional evaluation methods in a clinic, the attention of “efficacy” and “safety”, are declining, the causality between “gut microbiota”, “bacteria”, “nutrition”, and “abdominal pain” is attracting wide attention.

Using the diet or other manipulations of gut microbiota to modulate pain is not a primary focus of worldwide research, causing few papers (n = 1551, two keywords; n = 122, three keywords) to be discovered in this search. However, based on the real-world studies on patients with CD [44], DSPN [8], and even epilepsy (an unpublished study), we do find the benefits in alleviating pain when combining nutrition and microbiota as an integrated treatment plan. Based on the research strategy in WoSCC, the pain mentioned in this article is different from discomfort. Pain is more specific and of clinical significance than discomfort for physicians and researchers to pay attention to. Mounting preclinical and clinical evidence strongly supports the critical involvement of gut microbiota in visceral pain, inflammatory pain, neuropathic pain, and even headache by attenuating pain hypersensitivity (partially via a TRPV1-mediated mechanism) [11,35,45,46,47,48]. As the two clusters of the basic themes, both probiotics and prebiotics may represent a novel strategy for chronic pain management by targeting the gut microbiota. Probiotics such as *Bifidobacterium*, *Lactobacillus* genera (also presented in the motor themes) [49], and *Akkermansia muciniphila* (*A. muciniphila*) [50,51,52] are widely used in a clinic and in basic research related to pain improvement. Indeed, Cryan JF and Dinan TG suggested that probiotics play a role in improving pain in animals, and preliminarily revealed the mechanism of the microbiota–gut–brain axis [24]. Verdu et al. revealed that specific probiotic therapy attenuates antibiotic-induced visceral hypersensitivity in mice through modulating sensory neurotransmitter content (substance P) in the colon and altering visceral perception [29], while Tang et al. stated that SCFAs (produced with the bacterial fermentation of dietary fibers in the gut) are implicated in the modulation of chronic pain through several possible mechanisms [53]. Recently, Rebeca M et al. revealed that improvement in abdominal pain was associated with the relative abundance of *A. muciniphila* in IBS patients through FMT [54], mainly because *A. muciniphila* is a promising next-generation probiotic that produces multiple SCFAs as end products by degrading mucus [55]. As a factor to enhance the function of probiotics, the definition of prebiotic was put forward in 1995 [56] and modified in 2017 to denote a substrate selectively utilized by host microorganisms conferring a health benefit [57]. Generally, prebiotics include fructooligosaccharides (FOS), galactooligosaccharides (GOS), inulin, lactulose, polydextrose, and other mixtures of different components, which are nowadays generally added to food as a nutritional supplement. Prebiotics can increase resistance to pathogens, the regulation of immunity, mineral absorption, and healthy intestinal function, as well as affecting metabolism and satiety. Researchers recommended that prebiotics only, or combined with probiotics, could be used as an intervention to relieve pain in various diseases for treating gastrointestinal and psychosocial health symptoms, from cancer [58] to functional bowel disorder [59,60], irritable bowel syndrome (IBS) [26,61,62], inflammatory bowel disease (IBD) [63], and constipation [64]. However, only 13 publications suggested prebiotics as a potential preventive and therapeutic approach to nutritional management in patients with chronic pain. Larger studies should address how prebiotics modulate pain through gut microbiota.

The concept of fecal microbiota transplantation (FMT) involves transferring the whole gut microbiota and their products derived from healthy donor feces to restore healthy microbiota and function in patients with dysbiosis-related diseases [65]. We find that there were far more studies focusing on the applications of probiotics (single or combined) (200/1551) than of FMT (58/1551). One possible reason is that functional studies of single or several combined strains are easy to carry out in animal experiments or clinical research, while the mechanisms underlying the whole gut microbiota are difficult to explain. From the perspective of using microbial cells to treat diseases, probiotics and FMT are at two extremes. On the other hand, as the most anticipated gut-microbiota-based therapeutics in the last 10 years, the clinical value of FMT in relieving pain remains to be noticed and discovered. There is growing evidence that interaction between gut dysbiosis and malnutrition plays an important role at different stages of life, so targeting the whole gut microbiota via FMT may prevent or treat malnutrition [66,67]. In a clinic, FMT has already successfully treated recurrent *Clostridioides difficile* infection [68] and has shown mixed success in treating other conditions such as IBD, IBS, metabolic disease, and even cancer [44,69,70,71,72,73,74]. In 2015, our team reported that the rapid control and maintenance of abdominal pain after FMT treatment was a major feature in refractory CD [73]. Then, in 2021, abdominal pain as one of the seven therapeutic targets of CD was analyzed and found to be significantly improved at 1 month (72.7%), 3 months (70.5%), and 12 months (61.9%) in a large cohort study, respectively [44]. Moreover, other population and animal studies have also reported that FMT relieves pain by changing the composition of gut microbiota and the metabolites in patients with IBS and IBD, or regulating gene expression and immune cells in the peripheral nervous system to relieve neuropathic pain [14,75,76]. The therapeutic effects of FMT on chronic pain may include several mechanisms, such as direct competition between pathogenic and commensal bacteria, the restoration of secondary bile acid metabolism, and the protection and stimulation of the intestinal barrier and immune system. The combination of FMT and nutrition intervention such as enteral nutrition, parenteral nutrition, daily diet management, etc., may regulate gut microbiota to a better degree and improve the efficacy. Therefore, FMT may become a promising approach to the treatment of chronic pain, especially visceral pain related to GI disorders.

Dietary management is also considered in the thematic map; approximately 75% (91/122) of publications referring to how nutrition can modulate the pain–gut-microbiota axis pay attention to diet. Dietary modifications represent an important baseline and non-pharmacological therapy option for almost all clinical diseases, which is the most direct and effective source of nutrition. Various foods and diets are supported by a growing number of biological mechanisms as potential means to prevent and reduce chronic pain [77]. Especially those foods that have an anti-inflammatory, pro-resolving, or analgesic (anti-nociceptive) effect may play a role in the prevention and management of chronic pain [78]. Foods and diets that have been applied in clinical practice include oily fish, marine omega-3 fatty acids, the low-FODMAP (fermentable oligosaccharides, disaccharides, monosaccharides, and polyols) diet, and the combination of wheat peptides and fucoidan (WPF).

The role of gut microbiota in pain and the related perception is an area worthy of in-depth research. Its plasticity makes it an important factor in influencing the host’s physiological state, which may further affect pain. Geographical migration and changes of living habits may have an impact on gut microbiota, thereby influencing pain sensitivity. However, further studies are needed to exploit safer and more effective applications for a clinic. Interaction between nutrition, gut microbiota, and pain is bidirectional; a chronic pain condition can affect microbiota and nutritional status, while precise nutrition and microbiota intervention strategies can directly or indirectly affect pain through endocrine, immune, and neural systems. In a word, therapeutic implications such as probiotics, prebiotics, FMT, food, and dietary supplements are targeting the relationship between gut microbiota and nutrition in chronic pain conditions.

### 4.2. Limitations

This study has some limitations. Firstly, the data analyzed were only taken from the database of the WoSCC, potentially leading to source bias. However, it would be impossible to merge data from multiple databases as different databases record citation counts differently. Secondly, the data collected from the WoSCC were restricted by the information submitted by the authors and journals, resulting in information missing at random. Finally, some progress of the study was limited by the COVID-19 pandemic, which may have contributed a hysteretic quality to some degree.

## 5. Conclusions

Based on the bibliometric analysis and visualization of the literature, to some extent, we can know that the relationship between gut microbiota, pain, and nutrition is dynamic and complex, rather than only one of simple cause–effect. Studies that attempt to explore whether microbiota dysbiosis and the ensuing malnutrition is truly causative or merely a consequence of various pain in humans have suffered from several limitations, making it difficult to have definitive conclusions. More high-quality, mechanistic studies are warranted in clinical practice to explore molecular clues, particularly on the crosstalk between gut microbiota, nutrition, and pain.

## Figures and Tables

**Table 1:** The 10 most productive authors of research.

Section 1. Researchers on Pain–Gut-Microbiota Relationship				
Author	Number of Publications, n (%)	Citations per Publication (CPP)	H-Index ^a^	M-Index (First Year) ^a,b^
Cryan JF	28 (1.81)	234.21	22	1.47 (2009)
Dinan TG	23 (1.48)	261.78	21	1.4 (2009)
Mayer EA	16 (1.03)	211.13	13	0.87 (2009)
Zhang L	15 (0.97)	35.93	10	1.25 (2016)
Zhang Y	15 (0.97)	11	8	1.33 (2018)
Talley NJ	13 (0.84)	69.38	13	0.93 (2010)
Simren M	13 (0.84)	74	9	0.75 (2012)
Shulman RJ	12 (0.77)	103.17	10	0.77 (2011)
Bercik P	12 (0.77)	44.25	9	0.5 (2006)
Clarke G	11 (0.71)	225.45	10	0.67 (2009)
**Section 2. Researchers on Pain–Gut-Microbiota–Nutrition Relationship**				
**Author**	**Number of Publications, n (%)**	**Citations per Publication (CPP)**	**H-Index ^a^**	**M-Index (First Year) ^a,b^**
Simren M	5 (4.10)	123.40	4	0.36 (2013)
Mayer EA	3 (2.46)	203.00	3	0.27 (2013)
Li L	3 (2.46)	131.67	3	0.43 (2017)
Van Oudenhove L	3 (2.46)	17.67	3	0.50 (2018)
Bercik P	3 (2.46)	3.33	2	0.50 (2020)
Chung YM	2 (1.64)	187.50	2	0.29 (2017)
Hazen Sl	2 (1.64)	187.50	2	0.29 (2017)
Luscher TF	2 (1.64)	187.50	2	0.29 (2017)
Mach F	2 (1.64)	187.50	2	0.29 (2017)
Matter CM	2 (1.64)	187.50	2	0.29 (2017)

^a^ Calculated from the dataset. ^b^ Calculated by dividing the H-index by the number of years since the first published paper (within the dataset) of the author.

**Table 2:** The 10 most productive organizations of research.

Section 1. Organizations Regarding Pain–Gut-Microbiota Relationship				
Organization	Number of Publications, n (%)	Citations per Publication (CPP)	H-Index ^a^	M-Index (First Year) ^a,b^
Mayo Clinic	31 (2.00)	81.13	23	1.77 (2010)
University of California, Los Angeles	29 (1.87)	143.34	20	1.43 (2009)
Harvard Medical School	25 (1.61)	46.52	18	3.00 (2017)
McMaster University	25 (1.61)	81.60	16	0.94 (2006)
University of Washington	24 (1.55)	27.54	13	1.30 (2013)
University College Cork	23 (1.48)	51.26	11	0.73 (2008)
Baylor College of Medicine	20 (1.29)	83.25	12	1.00 (2011)
University of Gothenburg	20 (1.29)	50.35	10	0.91 (2012)
University of North Carolina	20 (1.29)	67.90	12	1.00 (2011)
Nanjing Medical University	19 (1.23)	33.00	12	1.50 (2015)
**Section 2. Organizations Regarding Pain–Gut-Microbiota–Nutrition Relationship**				
**Organization**	**Number of Publications, n (%)**	**Citations per Publication (CPP)**	**H-Index ^a^**	**M-Index (First year) ^a,b^**
University of Gothenburg	7 (5.74)	91.43	4	0.40 (2013)
King’s College London	4 (3.28)	39.75	4	0.33 (2011)
University College Cork	4 (3.28)	13.50	4	1.00 (2019)
Icahn School of Medicine at Mount Sinai	3 (2.46)	53.33	3	0.50 (2017)
Katholieke Universiteit Leuven	3 (2.46)	23.67	2	0.29 (2016)
Lerner Research Institute	3 (2.46)	126.33	3	0.50 (2017)
McGill University	3 (2.46)	8.33	2	0.29 (2016)
McMaster University	3 (2.46)	3.33	2	0.67 (2020)
Monash University	3 (2.46)	27.33	2	0.29 (2016)
Queen’s University	3 (2.46)	3.33	2	0.67 (2020)

^a^ Calculated from the dataset. ^b^ Calculated by dividing the H-index by the number of years since the first published paper (within the dataset) of the organization.

**Table 3:** The 10 most productive countries of research.

Section 1. Countries Regarding Pain–Gut-Microbiota relationship				
Country	Number of Publications, n (%)	Citations per Publication (CPP)	SCP ^a^	MCP ^b^ (MCP/(SCP+MCP) ^c^, %)
United States	356 (22.95)	48.90	287	69 (19.38)
China	283 (18.25)	15.40	251	32 (11.31)
Italy	119 (7.67)	24.10	86	33 (27.73)
Australia	58 (3.74)	27.70	39	19 (32.76)
Canada	55 (3.55)	35.40	33	22 (40.00)
United Kingdom	55 (3.55)	55.80	33	22 (40.00)
France	48 (3.09)	31.80	31	17 (35.42)
South Korea	44 (2.84)	20.10	40	4 (9.09)
Spain	44 (2.84)	35.10	31	13 (29.55)
Germany	42 (2.71)	39.10	32	10 (23.81)
**Section 2. Countries Regarding Pain–Gut-Microbiota–Nutrition Relationship**				
**Country**	**Number of Publications, n (%)**	**Citations per Publication (CPP)**	**SCP ^a^**	**MCP ^b^ (MCP/(SCP+MCP) ^c^, %)**
United States	31 (25.41)	44.2	22	9 (29.03)
Italy	17 (13.93)	12.4	10	7 (41.18)
China	9 (7.38)	20.0	5	4 (44.44)
Australia	7 (5.74)	6.7	6	1 (14.29)
Canada	7 (5.74)	4.7	5	2 (28.57)
Germany	7 (5.74)	87.7	4	3 (42.86)
Brazil	6 (4.92)	15.3	6	0 (0)
Belgium	4 (3.28)	8.5	0	4 (100)
Spain	4 (3.28)	13.0	4	0 (0)
United Kingdom	4 (3.28)	41.0	2	2 (50.00)

^a^ SCP: single-country publication. ^b^ MCP: multiple-country publication. ^c^ SCP and MCP were calculated with Bibliometrix based on data from the corresponding author’s country only. Hence, their summation did not equal the total number of publications of that country.

**Table 4:** The 10 most productive journals of research.

Section 1. Journals Regarding Pain–Gut-Microbiota Relationship						
Journal	Number of Publications, n (%)	Citations per Publication (CPP)	H-Index ^a^	M-Index (First Year) ^a,b^	2023 Impact Factor	Research Area (Domain)
Nutrients	46 (2.97)	17.52	16	1.60 (2014)	5.9	Medicine
World Journal of Gastroenterology	38 (2.45)	44.76	19	1.27 (2009)	4.3	Medicine
Neurogastroenterology and Motility	31 (2.00)	36.74	18	1.29 (2010)	3.5	Medicine
PLoS ONE	26 (1.68)	29.19	14	1.08 (2011)	3.7	Multidisciplinary Sciences
Scientific Reports	25 (1.61)	35.92	15	1.67 (2015)	4.6	Multidisciplinary Sciences
Frontiers in Cellular and Infection Microbiology	22 (1.42)	11.27	8	1.14 (2017)	5.7	Medicine
International Journal of Molecular Sciences	21 (1.35)	15.48	10	1.43 (2017)	5.6	Chemistry
Medicine	20 (1.29)	11.20	7	0.78 (2015)	1.6	Medicine
Gastroenterology	19 (1.23)	104.63	18	1.38 (2011)	29.4	Medicine
Frontiers in Microbiology	16 (1.03)	11.25	7	0.54 (2011)	5.2	Microbiology
**Section 2. Journals Regarding Pain–Gut-Microbiota–Nutrition Relationship**						
**Journal**	**Number of Publications, n (%)**	**Citations per Publication (CPP)**	**H-Index ^a^**	**M-Index (First Year) ^a,b^**	**2023 Impact Factor**	**Research Area (Domain)**
Nutrients	11 (9.02)	20.55	5	0.56 (2015)	5.9	Medicine
World Journal of Gastroenterology	5 (4.1)	19.20	4	0.36 (2013)	4.3	Medicine
Animals	3 (2.46)	10.33	3	1.00 (2021)	3.0	Medicine
Nutrition in Clinical Practice	3 (2.46)	26.67	2	0.18 (2013)	3.1	Medicine
American Journal of Gastroenterology	2 (1.64)	17.50	2	0.18 (2013)	9.8	Medicine
Autoimmunity Reviews	2 (1.64)	32.50	2	0.25 (2016)	13.6	Medicine
BMJ Open	2 (1.64)	2.00	2	0.50 (2020)	2.9	Medicine
Clinical Gastroenterology and Hepatology	2 (1.64)	36.50	2	0.40 (2019)	12.6	Medicine
Clinical Nutrition	2 (1.64)	37.00	2	0.18 (2013)	6.3	Medicine
European Heart Journal	2 (1.64)	187.50	2	0.29 (2017)	39.3	Medicine

^a^ Calculated from the dataset. ^b^ Calculated by dividing the H-index by the number of years since the first published paper (within the dataset) of the journal.

**Table 5:** The 10 most locally cited studies of research.

Section 1. Literature Regarding Pain–Gut-Microbiota Relationship						
DOI	Author	Year	Journal	LC	GC	LC/GC (%)
10.1038/nrn3346	Cryan JF [24]	2012	Nature Reviews Neuroscience	102	2385	4.28
10.1056/NEJMoa1004409	Pimentel M [28]	2011	New England Journal of Medicine	85	665	12.78
10.1136/gut.2005.066100	Verdu EF [29]	2006	Gut	78	307	25.41
10.1053/j.gastro.2011.06.072	Saulnier DM [30]	2011	Gastroenterology	64	440	14.55
10.1038/nrgastro.2009.35	Rhee SH [31]	2009	Nature Reviews Gastroenterology & Hepatology	63	766	8.22
10.1111/nmo.12103	Crouzet L [25]	2013	Neurogastroenterology & Motility	58	176	32.95
10.1136/gut.2008.167270	Moayyedi P [32]	2010	Gut	57	461	12.36
10.1038/ajg.2014.202	Ford AC [33]	2014	American Journal of Gastroenterology	56	456	12.28
10.1016/j.neuroscience.2014.07.054	O’mahony SM [34]	2014	Neuroscience	55	179	30.73
10.1073/pnas.0711891105	Amaral FA [35]	2008	Proceedings of the National Academy of Sciences of The United States of America	54	173	31.21
**Section 2. Literature Regarding Pain–Gut-Microbiota–Nutrition Relationship**						
**DOI**	**Author**	**Year**	**Journal**	**LC**	**GC**	**LC/GC (%)**
10.1038/nrdp.2016.14	Enck P [26]	2016	Nature Reviews Disease Primers	3	514	0.58
10.1016/j.jand.2020.08.077	Croisier E [27]	2021	Journal of the Academy of Nutrition and Dietetics	2	7	28.57
10.2174/156652408784533779	Lutgendorff F [36]	2008	Current Molecular Medicine	1	127	0.79
10.1016/j.clnu.2012.08.010	Waitzberg Dl [37]	2013	Clinical Nutrition	1	54	1.85
10.1038/ajg.2013.75	Le Neve B [38]	2013	American Journal of Gastroenterology	1	31	3.23
10.1016/j.pmr.2014.12.006	Tick H [39]	2015	Physical Medicine and Rehabilitation Clinics of North America	1	21	4.76
10.3390/nu7095380	Deng Yy [40]	2015	Nutrients	1	168	0.60
10.1097/NCC.0000000000000286	Kelly Dl [41]	2016	Cancer Nursing	1	10	10.00
10.1016/j.gastrohep.2015.07.009	Molina-Infante J [42]	2016	Gastroenterologia Y Hepatologia	1	22	4.55
10.1080/17474124.2017.1359539	Wegh Cam [43]	2017	Expert Review of Gastroenterology & Hepatology	1	32	3.13

LC: local citations; GC: global citations.

## Data Availability

Datasets generated for this study are included in the article. Additional data and materials underlying this article will be shared on reasonable request to the corresponding author.

## Abbreviations

CD	Crohn’s disease
DM	Diabetes mellitus
DSPN	Distal symmetric polyneuropathy
FMT	Fecal microbiota transplantation
SCFAs	Short-chain fatty acids
BAs	Bile acids
WoSCC	Web of Science Core Collection
NP	Number of publications
NC	Number of citations
CPP	Citations per publication
LC	Local citations score
GC	Global citations score
SCP	Single-country publication
MCP	Multiple-country publication
APY	Average published year
FOS	Fructooligosaccharides
GOS	Galactooligosaccharides
IBS	Irritable bowel syndrome
IBD	Inflammatory bowel disease
FODMAP	Fermentable oligosaccharides, disaccharides, monosaccharides, and polyols
WPF	Wheat peptides and fucoidan
